# The Impact of Early Robotics Education on Students’ Understanding of Coding, Robotics Design, and Interest in Computing Careers

**DOI:** 10.3390/s23239335

**Published:** 2023-11-22

**Authors:** Gisele Ragusa, Lilian Leung

**Affiliations:** Viterbi School of Engineering, Division of Engineering Education, University of Southern California, Los Angeles, CA 90007, USA; lilianl@usc.edu

**Keywords:** early robotics, coding education, computer career aspirations, robotics learning

## Abstract

Early robotics education has been sparsely researched, especially for children in elementary education. This research pertains to an early education study that introduced robotics design and programming to children in early education with the purpose of increasing their robotics design knowledge, improving their coding skills, and inspiring their aspirations for future careers. It represents a seven-year study of students ages seven through ten years in a large urban school district. The study engaged a pre–post program comparison of the robotics and coding intervention that focused on children’s improved understanding of robotics in addition to their career aspirations. The study resulted in increases in the participating students’ understanding of robotics design as well as improved coding skills in robotics contexts. Furthermore, the study also led to increases in the students’ career aspirations toward computing fields.

## 1. Introduction

Engaging educational programs that involve science, technology, engineering and mathematics (STEM) with young children are of critical importance because such a curriculum is often underdeveloped in early childhood education [1]. Research has determined that incorporating educational robotics into young children’s educational experiences leads to significant improvement in their engineering and technological conceptual understanding and assists them in engaging in computational thinking and in developing their technology understanding. Robotics in particular also helps young learners to engage in problem solving [2,3,4,5]. This manuscript presents the results of one such impactful program, a robotics and coding academy, that had as its primary purpose the goal of alleviating the relatively sparse availability of STEM programs in elementary education to inspire young learners to learn to design and program robots and to consider future computing careers.

### 1.1. The Importance of Integrating Educational Robotics into Early Childhood Education

Integrating educational robotics into early education is not only an engaging and entertaining way to incorporate STEM into education, robotics education has also demonstrated significant impacts on children’s computational thinking and their ability to identify and solve problems [2,4,5]. Computational thinking is most often measured in three dimensions, all of which correspond to components of robotics education, especially when programming is included: sequence, action instruction correspondence, and debugging. More specifically, sequences test if students can respond to a challenge through programming activities; action–instruction correspondence determines if children can relate the instruction provided in robotics education to the performance of the robots they are programming; and debugging is the ability that students have to identify problems and to correct programming errors (a proxy for problem-solving) [4]. Robotics educational programs can demonstrate that young children are able to develop the skills and capacity to problem solve using programming languages and the fundamentals of computational science. Educational robotics can provide a means for “leveling” the playing field in early STEM education that is both engaging and meaningful for diverse children with varying experiences, resources, and backgrounds. Educational robotics provides a variety of learning opportunities and experiences in STEM and increases children’s confidence in their abilities to apply computational skills and participate in a rapidly changing technological world [5]. Furthermore, robotics education enables students to develop abstract thinking and a positive attitude toward STEM learning and future STEM career aspirations at an early age. It also facilitates children’s ability to express themselves using technological tools, think critically, and design innovatively. Through robotics, students are able to experiment, design, identify, and solve problems (correct errors) through active learning pedagogies which facilitate constructionist learning [2,6]. Such educational programs create opportunities for children to explore basic technical concepts, experiment with technology, and design and build robots to solve problems. Educational robotics programs also promote teamwork and active communication between peers and their teachers. Through back-and-forth dialogue and bouncing ideas off of one another, students are able to reflect on and communicate their shared understanding of the role of technology in problem-solving. Accordingly, project and inquiry-based pedagogy, which is most commonly applied in educational robotics, not only facilitates children’s problem-solving practices but also provides students with early opportunities to engage in innovation and engineering design practices.

### 1.2. Educational Robotics Program Impacts in Early Childhood

Robotics education remains relatively sparse for the youngest of learners; however, several robotics programs have been recently implemented in school settings, and research is emerging measuring their effectiveness and impact. There are some programs, however, with a focus on improving children’s understanding of robotics and coding in pre-college education. One such robotics and coding educational program is the Bee-Bots program, which enables young students to program and control the movements of bee-shaped robots by programming them using directional commands. Cubelets devices, used in this particular educational robotics program, encourage students to build a sensored robot and utilize applications from cube-shaped magnetic robotic blocks.

Similarly, Muñoz-Repiso and Caballero-González studied the impact of this program on 131 children (ages 3–6) at a Spanish school in Salamanca [5]. The program, entitled TangibleK, used the described Bee-Bot robotics kit to engage students in experimentation, design learning, and error correction through active learning. Muñoz-Repiso and Caballero-González sought to determine if it was possible to develop computational thinking in young children through robotic activities while simultaneously improving children’s ability to sequence actions and correct programming errors [5]. These researchers determined that the children involved in the TangibleK intervention program were able to think computationally and engage in programming sequencing. The learning gains for those involved in the program were significantly higher than for those not participating in the program.

In a related program, Castro and colleagues studied the impact of early educational robotics through a program called RoboticsEd [4]. This particular robotics program used a range of developmentally designed robots. The researchers sought to test the impact of the program across age levels with children ages 7–14 using a pre–post comparison approach. The results of this research indicated significant improvement in each age group assessed between the pre- and post-program periods (*p* = 0.000 across age groups). Furthermore, there were no gender differences in performance on the pre–post assessment tasks (7–9 years old: *p* = 0.307, 9–11 years old: *p* = 0.842, and 11–14 years old: *p* = 0.219) for the research.

In a study conducted by Zviel-Girshin, Luria, and Shaham, 83 kindergarteners and 113 first-graders participated in an early robotics education program designed to foster the integration of robotics as part of science and technology courses to allow children to construct, program, and play with robots [6]. The authors sought to determine if there were gender and age differences in the children’s beliefs about their ability to construct a new and/or complex robotic model as well as to determine the children’s ongoing understanding of the basic principles of robotics, usage of sensors, programming, and problem-solving after engaging in an educational robotics program. The results of this study revealed that 59% of boys versus 46% of girls and 58% of kindergarten children versus 48% of elementary school children demonstrated more confidence when constructing a new and complex robotic model. Such understanding of robotics skills was found to persist beyond the scope of the robotics education program.

Children with special needs have also been studied comparatively in educational robotics programs. In a study conducted by Warren and colleagues, young children with autism spectrum disorder completed an intervention in which they interacted with a humanoid robot and a human social partner to complete a series of motor movements. Warren and colleagues sought to test out the hypothesis that children with autism spectrum disorder would demonstrate increased attention to the robot during the sessions compared to a human counterpart by assessing the children’s imitation skills [7]. These researchers used sensor data and gesture recognition while children with autism spectrum disorder raised one or two hands, waved, or reached their arms out to the side and compared the percentage of time they engaged in looking at a human versus a humanoid robot. The results of this study indicated that children with autism spectrum disorder demonstrated higher imitation performance with the humanoid robot than they did with the human administrator (38%). This study demonstrates the relatively universal keen interest that children have in robots.

### 1.3. Coding and Its Impact on Child Learning

Although coding has been traditionally taught in higher education, educational programs have begun to integrate coding into K-12 over the last decade. This, however, is more sparsely applied to the education of children under the age of eight. Many countries, especially in Europe, have begun to offer coding to young children as early as kindergarten (ages 5–6). Although the United States is progressing in providing coding in the K-12 curriculum as a requirement, such structures are not yet universally in place in the U.S. K-12 curriculum. There are, however, kindergarten and elementary school teachers who voluntarily implement coding and programming curricula in their classrooms. One of the challenges in teaching coding in the U.S. is the teachers’ limited coding knowledge in addition to their limits in coding education resources. More than 75% of U.S. teachers who educate children under the age of ten have little or no experience in coding before integrating coding into their classrooms [8]. Although teachers often do not have high confidence in their coding abilities, they make brave attempts to incorporate it into their curriculum as they consider coding a gateway to better future career opportunities for their students. Furthermore, some teachers view coding education as a means of developing their students’ learning mindsets, resilience, confidence, and motivation. They also recognize that coding may enhance children’s ability to problem solve, engage in logical thinking, and practice analytical reasoning.

### 1.4. Programming Languages across Grade Levels

One of the most common programming languages that is currently taught to young children globally is Scratch, which is followed by Blocky, Python, and JavaScript. Scratch is typically introduced at the lower primary grade levels in K-12 education as a “drag-and-drop” opportunity for young learners. Developmentally, it is often transitioned to practice with Python and JavaScript for secondary grade children. This developmental transition of learning programming languages enables young children to progress from visual to textual language as they learn to code and prevents them from relying solely on block-based computing language. As an example of this, in a study by Price and Price -Mohr which focused on teaching text-based language (Java), children were taught programming language between ages three and six by having them write and code an animated story [9]. These researchers evaluated how children compose their code and observed their purposeful code corrections. Using text-based coding language, the children in the study demonstrated the importance of syntax and made better use of various programming functions at higher cognitive levels.

Bers purports that coding as another language, an increasingly common way to teach computer coding to young children, can be used as an impactful pedagogical approach to teaching coding [10]. The coding as another language perspective views teaching coding as a way of expanding children’s literacy (and in this case computer literacy), by teaching a variety of languages, such as programming language, while enhancing children’s skills in explanation, argumentation, and open-ended interpretation in computer science. The goal of coding as another language pedagogy is to facilitate a successful transition through the six coding stages: emergent, coding and decoding, fluency, new knowledge, multiple perspectives, and purposefulness. The six coding stages are adapted from traditional literacy education and repurposed for computer programming. Using this pedagogical approach, at the emergent level, children are offered the opportunity to explore hardware (a tablet) and software (ScratchJr app, Version 3), observe and identify different basic elements of coding from teachers’ demonstrations, and create simple programming designs. Just as they are with traditional literacy instruction, children are given a storybook to look at and practice reading. At the coding and decoding stage in coding as another language instruction, children learn about syntax and the correlation between their choices of codes and the impact on technology (robots and other tech.) They begin to engage in problem-solving and debugging while expressing themselves by creating their personal story [10]. Chevalier and colleagues underscore [11] these efforts in their discussion of computational thinking-related pedagogical approaches. Similar to when children begin to learn how to read and write traditionally, children are given the opportunity to read and analyze the “story”. As they move along the coding “ladder”, children are not only learning to code, but they are also coding to learn. Through trial and error, they apply their emergent coding knowledge in sequencing and algorithmic thinking as a means of learning new mathematical skills. They discover and gain new knowledge and perspectives about coding as a language. Similar to how children gradually learn more difficult words and interpret texts differently as they are exposed to more books (especially in informational text), their coding understanding and use progress developmentally. The pathway of stages of coding as another language is non-linear. In other words, students go back and forth to develop and master coding knowledge. Coding as another language pedagogy acknowledges that the strategies used in traditional literacy education can be applied to teaching students coding language, skills, and strategies. It allows young children to practice their sense-making, self-expression, and communication in computer science in early childhood just as they do with traditional language and literacy development, using a developmental approach.

An advantage of teaching coding to young children is that it can easily be integrated into different subjects such as mathematics, science, engineering, and language arts. As previously described, the most common programming application for young children, ScratchJr, integrates coding into literacy through a story-writing–coding approach. Children learn to use the concepts of abstraction, decomposition, logical thinking, and patterning in both their story writing and their coding processes [9]. It also enhances skills in explanation, argumentation, and open-ended interpretation in computer science [10]. There are several educational applications that are designed to enable students to learn coding and develop literacy simultaneously. Applications such as ScratchJr. And Kodable encourage children to express themselves by creating a story and animating it through coding [12,13]. Some coding applications are developed to enable children to learn sequencing and if-then commands. Integrating applications allow students to see that visual programming language, enhances computational thinking, and encourages students to practice logic, reasoning, and problem-solving, in addition to literacy concepts such as decomposition, abstraction, narrative schemas, logical thinking, and patterning. These practices underscore that when coding as another language teaching pedagogy is personal and relevant to children, it provides purposes and motivations to learn about coding and to use it.

Robotics and coding learning tools such as Code-a-pillar, Bee-Bot, Kibo, Kubo, Makey Makey, and Scratch can be easily implemented in the classroom for robotics paired with learning to code as these opportunities are intuitive, subject-agnostic, and open-ended. Children can explore and master the basic programming functions independently or with sparse assistance. Rather than teaching coding as a separate subject in early education, these opportunities enable teachers of young children to integrate coding into the existing curriculum using their existing curricular standards. These hands-on learning opportunities encourage children to explore, discover, and experiment as they become learners, thinkers, and future innovators [14].

There is vast potential in teaching and integrating robotics and coding in early childhood education. This is represented in work by Chalmers [15], the pedagogical research of Chiu [16], and in the extensive review of the literature in the areas of computational thinking by Yang and colleagues [17]. This review of the literature supported the design and implementation of the study that is presented in this manuscript, which is an example of one such integrated early robotics education opportunity, the Robotics and Coding Academy (RCA). The RCA has as its primary goal the aim of teaching children to design and program robots with an aim of inspiring young children to pursue robotics, computer science, and other technologically focused majors in college and their eventual careers. The program is developmentally appropriate, as described by best practice in the review of pertinent research (above), and has not only been able to improve its participating students’ robotics design understanding and increase their coding skills but has also been able to increase participating students’ early career aspiration toward computer science and particularly robotics.

## 2. Materials and Methods

The Robotics and Coding Academy (RCA) is an afterschool computer science intervention program offered by a research university that has as its primary purpose the goal of teaching children ages seven through ten to design and program robots. It targets urban elementary school students in grades three and four who come from under-resourced city-dwelling families in the western United States. RCA is intended not only to teach robotics design and coding skills; it is also designed to inspire young learners to consider computer science majors in college and ultimately to create an aspirational career pathway in robotics and other computing careers.

**Figure 1:**
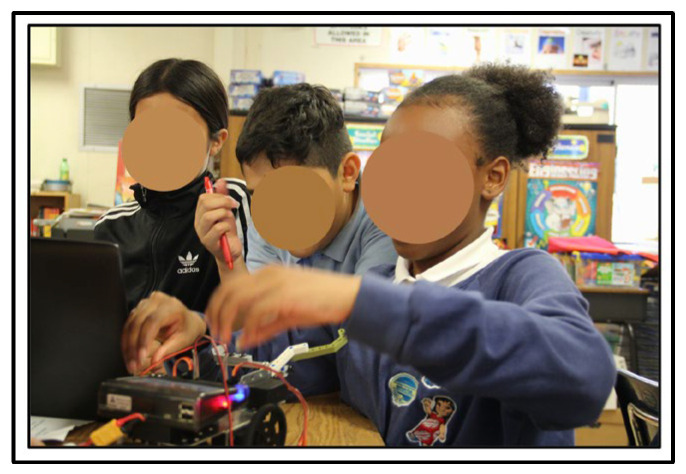
Children design and program a robot (photo de-identified).

The RCA program has been in operation for seven years, and 862 students have attended the program. The participating children are joined by a university faculty member and their third- and fourth-grade teachers in addition to ten–fifteen university student near-peer mentors. The classrooms in which RCA is offered are situated in five large urban schools in the second-largest school district in the United States. Approximately 92.8% of the children participating in the RCA come from financially under-resourced (low-income) families, and less than 35% of these families have technology at home other than a television and a smartphone. Approximately 53% of the participating students are girls, with the remaining 47% of participants being boys. The gender parity in student recruitment for the RCA program was deliberate. The students’ ethnicities in the RCA program were 43% Hispanic/Latinx, 28% African American/Black, and 13% White/European American, 4% Asian/Pacific Islander, and the remaining children had mixed ethnicities according to child self-report. For all participating students, this was their only experience in a robotics or coding educational program, although 15% participated in the program for two years consecutively. Such recruitment demographics were selected by the children‘s teachers and the diversity-focused and gender parity recruitment target was deliberate as it mirrors urban school children in the western US. An example of such diversity is represented in Figure 1 (above). This research is fully approved by the university’s human subjects board and in particular, the review includes appropriateness and protection of minors.

In terms of the university student mentors in the RCA program, there was a 50–50 split in terms of gender with the students’ ethnicities matching the children’s socio-demographics. The mentors’ university majors were computer science, electrical engineering, mechanical engineering, and industrial and systems engineering with one–four university mentors per major. Prospective mentors were interviewed to participate in the project in the form of a standard job interview. The interviewers were university faculty members, and they assessed the prospective mentors’ backgrounds and experience with children during the interview. The mentors were matched in terms of experience, socio-demographic similarity, and experience with children. The mentors received training and certification for working with children prior to beginning participation.

**Figure 2:**
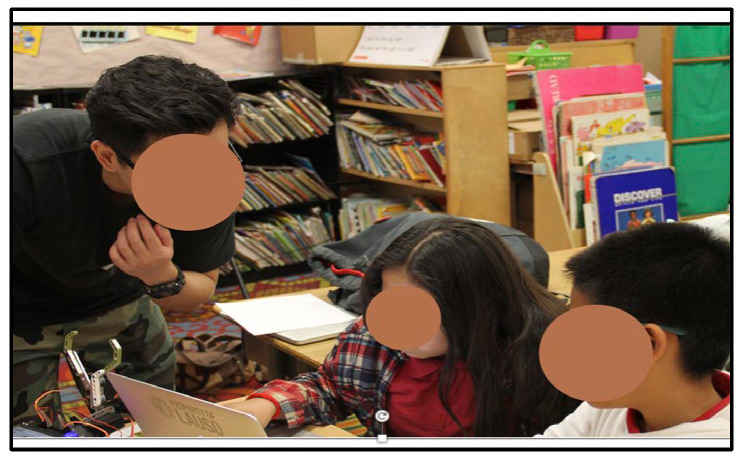
Children receiving robotics/coding mentorship from university students (photo de-identified).

The primary aims of the Robotics and Coding Academy intervention are to engage elementary-grade students with computer science and coding through robotics as a means of fostering students’ interest in computer science and to provide a pathway toward college by engaging them with rigorous, standards-aligned computer science curricula with university students as STEM tutors and near-peer role models. The program occurs after school for 1.5 h weekly for a total of 16 weeks per academic year. There are sixteen lessons taught to the students in teams of 4–5 students by a university student mentor. Each lesson uses a project-based learning pedagogical approach in which, depending on the lesson, the children complete a robotics, coding, or combination project during the lesson. The lessons proceed with the participating children reviewing the previous week’s lesson at the start of the lesson to activate their background knowledge. Then. the students are introduced to the project of the day, engage in demonstrations, participate in guided practice, and then complete the project of the day with their team. The children in the program learn to code in C to program their robot and are taught to design a robot using engineering design cycles and principles and then program a robot so that it can move forward and back, turn in circles, pick up items with a robotic arm, and “dance”, using the students’ free choice programming. The participating students engage (seen in Figure 2) in friendly school site competitions and showcases for their parents, thereby increasing their motivation to persist start-to-finish with the academic year curriculum.

The research involving the RCA program responds to four impact-focused research questions:

1. What is the impact of RCA participation on increasing children’s understanding of engineering design?
2. What is the impact of RCA participation on increasing children’s programming/coding skills?
3. What role does the RCA program have in inspiring its participants to major in computer science in college?
4. What role does the RCA program have in inspiring its participants to consider a career in computer science and/or specifically robotics?

**Figure 3:**
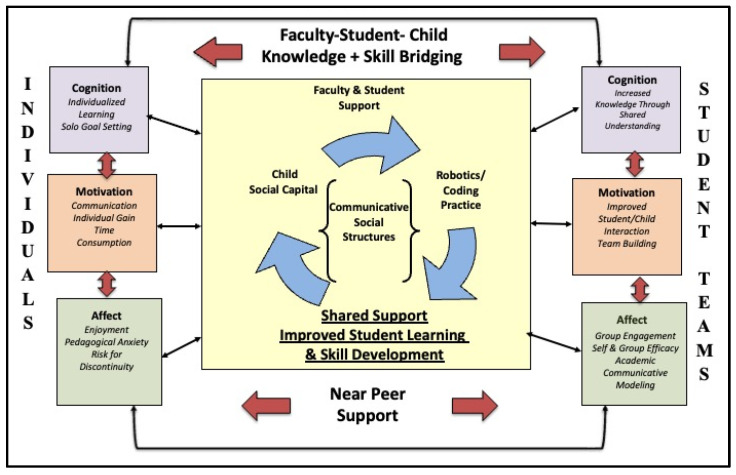
Robotics and coding academy mentorship model.

To respond to these four research questions, a combined developmental and socio-constructivist theoretical approach was used, meaning that the participating children work in robotics “teams” with near-peer mediation and mentoring as they learn coding skills and begin to understand robotics design principles. The developmental influence on the curriculum for the intervention necessitated that the children be taught to code in a way that is similar to traditional literacy development, building on the work of Bers’ coding as another language pedagogical approach [10]. Accordingly, the theory of action for this robotics and coding intervention is illustrated in Figure 3.

In terms of measuring the impacts of the Robotics and Coding Academy, the participating children’s robotics design and coding knowledge was assessed using a robotics and coding concept inventory, which contains forced-choice items about the nature of robotics design principles, and practice of coding and recognizing components as well as what occurs in computing and coding errors in robotics. More specifically, the students were asked to describe the robotics design cycle they used in building their robots and what their robots’ key features were. They were also asked to analyze small bits of code for errors comparatively. They were asked to determine the next steps in coding to initiate robotic arm clasps, as well as the forward and backward movement of the robots. This robotics and coding concept inventory was paired with a questionnaire that measures children’s motivation for computer science and career aspirations. In this career aspiration measure, the students were asked to rate items related to interest in coding, frequency of use of coding, and interest and frequency of use in building and designing items. Moreover, they were asked to rate themselves on the likelihood that they would become a computer scientist or roboticist and also go to college to study robotics and coding. Both the concept inventory and the career aspiration measure (described herein) were compared pre- and post-robotics and coding academy participation. The motivation and career aspiration measure is aligned with the learning and motivation theory of Mayer and colleagues. All of the research instruments were developed and tested for reliability and validity using item response theory building blocks for constructing measures [18,19]. For these program impact measures, a pre–post comparative statistical analysis using paired sample *t*-tests was employed [20].

## 3. Results

The results from the RCA program intervention have been interesting and impactful. Over the past seven years, the program has resulted in 67% gains in robotic design knowledge (26% pre-program versus 93% post) and 89% gains in coding skills and knowledge (3% pre-program versus 92% post) from the start to finish of the program combined for seven years. More specifically, the results of a paired sample *t*-test on changes in robotics design knowledge (pre-to-post) are t(862) = 2.591, *p* < 0.01. The results of a paired sample *t*-test on coding knowledge and skills (pre-to-post) are t(862) = 2.98, *p* < 0.001. Increases in career aspiration and motivation pre–post (sum scores) are 32%; t(859) = 1.41, *p* < 0.05. Importantly, 15% of the students enrolled in and completed the RCA program for two consecutive years, and therefore, comparisons across enrollment years were made. These cross-year comparisons indicate that the amount of time students spend in the program is positively correlated with their increases in coding knowledge, with statistical significance (r = 0.269, *p* < 0.05). These comparative results demonstrate the program’s positive impact both on student knowledge gained from the program and on improvements in career aspiration. Given the marked change in the understanding of robotics design and especially in coding, it is clear the program had impacts. This is most probably attributed to the fact that the children had no coding experience at all pre-program and very little experience if any at all in robotics or any engineering design-type programs. In terms of career aspiration, it was clear from the instrument responses pre-program that most of the children did not know what computer scientists and roboticists (and other STEM fields) did in terms of a profession. This clearly changed as they progressed throughout the RCA program.

## 4. Discussion

The empirical results of the Robotics and Coding Academy intervention demonstrate that even for young children, learning to design robots and program them enables them to learn a great deal of coding skills and to develop design understanding, both of which have been recognized as important 21st century skills in the United States [16]. The participants in this intervention program have also begun to understand the role of computer science and robotics in the workforce as demonstrated by their motivation and aspiration to consider computer science and robotics as a future career. While the researchers of the Robotics and Coding Academy intervention program recognize that many factors impact students’ learning and their aspirations for their future, given the limited resources that the children who participated in the program had in their homes, when paired with the uniqueness of the educational experience for these particular children, it is highly likely that the empirical results reported in this manuscript can be attributed to the intervention received by the children, especially since it has been in operation for seven years, and the results remain consistent year-by-year with optimal fidelity of its implementation.

### 4.1. Study Limitations

This study is limited by the students’ self-selection to participate in the program. Any child who wished to sign up for the program could sign up, with parental permission. Accordingly, the students may be more motivated than their non-participating peers in the program. The study is further limited by the fact that the researchers could not entirely infer that the results were a consequence of the program, especially since data were collected on whether the children had been a part of another robotics and or coding program, and none had, except for the 15% repeaters in the program. Accordingly, the researchers could not determine the full impact of the particular educational intervention without absolute certainty for two reasons: (1) they could not be certain of other educational exposure touched upon by the intervention and (2) the researchers could not be certain about other out of school experiences that students were exposed to and their relative impact on the research results. One final constraint or limitation of the program was the fact that it was limited by timing. The schools in which the program was provided required that it be once weekly, as a 1.5 h program to fit in with their school-based afterschool administrative constraints.

### 4.2. Practical Significance

This research has practical significance as it is a program that can be readily used in other young children’s classrooms. It uses off-the-shelf robotics, and if a school has a relationship with a university, it can partner with the university’s computer science or engineering departments or schools to engage in these efforts. It provides a highly applicable opportunity for young children to be exposed to robotics and coding prior to entering middle school, which is often the time in a child’s education when robotics is typically introduced.

## 5. Conclusions

This research underscores that working with young children in robotics and coding education can be helpful for them both in terms of the new knowledge they may gain and also in broadening their understanding of the importance and use of coding and how it works with robots. This can in turn inspire children to seek college majors and careers in computing. The presented research reinforces this impact and adds to the literature on the importance of introducing children to technology and technological “language” at an early age, during a developmental period in their lives when hands-on engagement with technology serves as a developmentally appropriate tool for learning and aspirational development. The program’s socio-constructivist approach falls in direct alignment with the RCA’s project-based approach of building and operating a robot within the lesson design and instructional delivery. Furthermore, the use of coding followed a developmental trajectory using CAL, as described in the manuscript’s review of the relevant literature.

## Data Availability

Data supporting the study’s reported results can be found under encrypted e-files on the primary author’s computer in data storage under lock and key in her office. The data are not publicly archived as minors were involved in the research. Masked data may be made available upon reasonable request with permission from the applicable parent or guardian.
